# Preliminary Molecular Survey of the Possible Presence of *Xylella fastidiosa* in the Upper Ionian Coasts of Calabria, Italy, through the Capture and Analysis of Its Main Vector Insects

**DOI:** 10.3390/insects12050446

**Published:** 2021-05-13

**Authors:** Luca Lombardo, Pierluigi Rizzo, Carmine Novellis, Veronica Vizzarri

**Affiliations:** 1 Department for Sustainable Food Process, Università Cattolica del Sacro Cuore, 29122 Piacenza, Italy; 2 CREA Research Centre for Olive, Citrus and Tree Fruit, 87036 Rende, Italy; rizzo.pierluigi@yahoo.com (P.R.); carminenovellis89@gmail.com (C.N.); veronica.vizzarri@crea.gov.it (V.V.)

**Keywords:** *Xylella fastidiosa*, Philaenus spumarius, Neophilaenus campestris, Calabria, olive orchards, meadows

## Abstract

**Simple Summary:**

As a consequence of the advancement of the outbreak front in Apulia (Italy), and the possibility of pathogenic insects being “hitchhiked” over long distances, the neighboring regions must implement a monitoring system for the control of the spittlebug vectors of *Xylella fastidiosa*. In this sense, the aim of this work was to evaluate the eventual presence of *X. fastidiosa* in olive orchards and meadows in the upper Ionian coasts of Calabria, Italy, through the capture and molecular analysis of its main vector insects.

**Abstract:**

*Xylella fastidiosa* subsp. *pauca*, strain CoDiRO is the bacterium responsible for the onset of the disease known as the olive quick decline syndrome, which has been causing a phytosanitary and economic emergency in the Apulia region since 2013. To date, three insect species have been identified as pathogenic carriers of *X. fastidiosa*. With the advancement of the infection front, and the possibility of pathogenic insects being “hitchhiked” over long distances, the monitoring of the vectors of *X. fastidiosa* in the Italian regions bordering Apulia is an increasingly contingent issue for the rapid containment of the bacterium and the protection of the olive-growing heritage. Accordingly, the present research concerned the capture and recognition of the vector insects of *X. fastidiosa* in the upper Ionian coasts of Calabria (Italy) to evaluate the possible presence of the bacterium through molecular diagnostic techniques. The sampling allowed us to ascertain the presence of *Philaenus spumarius* and *Neophilaenus campestris* and their preferential distribution in olive groves and meadows, whereas all the 563 individuals tested negative for the pathogen.

## 1. Introduction

*Xylella fastidiosa* is a non-spore-forming Gram-negative phytopathogenic bacterium belonging to the Xanthomonadaceae family, colonizing the xylem vessels of almost 600 plant species—275 genera and 85 families [1]. As such, it is the agent of a wide range of diseases, including Pierce’s disease of grape, citrus variegated chlorosis (CVC), peach phony disease, and plum leaf scald leaf scorch diseases on almond, coffee, and oleander [2].

Three subspecies have been formally accepted: subsp. *fastidiosa*, *pauca,* and *multiplex* [3], while three additional subspecies—*sandyi* (on *Nerium oleander*), *tashke* (on *Chitalpa tashkentensis*), and *morus* (on mulberry)—have been subsequently proposed. On the other hand, intra- and inter-subspecies recombination events have been also described [4,5,6]. Within the subsp. *pauca,* the strain CoDiRO (ST53) has been identified [7,8] as responsible for the olive quick decline syndrome (OQDS, equivalent of the Italian CoDiRO, complesso del disseccamento rapido dell’olivo), first observed in 2013 in the olive orchards of the Apulia region (Italy) and characterized by the extensive scorching of leaves and branches [9]. The plant-to-plant transmission mediated by xylem-sap feeding insect vectors led to the rapid spread of the pathogen, thus extending the affected area to approximately 750,000 hectares to date [10], with a spreading rate of about 20 km per year [11,12]. As a result, olive production in Apulia dropped from ~1,150,000 Mg in the 2006–2013 period, to ~805,500 Mg in the 2014–2020 period [13]. Although any xylem-sap feeding insect could theoretically transmit the bacterium, only three species (Hemiptera, Aphrophoridae) have been proven to be capable of acquiring the CoDiRO strain from infected olive plants and spreading it to other plants: *Philaenus spumarius*, *P. italosignus,* and *Neophilaenus campestris* [14]. *X. fastidiosa pauca* ST53 has also been found in the anterior intestines of some specimens of *Euscelis lineolatus* Brullé (Hemiptera, Cicadellidae). It was captured in two Apulian olive groves [15] in adult individuals of *Latilica tunetana* (Hemiptera, Issidae) during a transmission experiment to assess their ability to acquire the bacterium from infected plants [16], and in four (out of 314) specimens of *Cicada orni* L. (Hemiptera, Cicadidae) during a two-year experiment in an infected olive orchard of the region [17]. Nevertheless, their role in transmitting the bacterium to other olive plants has not been demonstrated.

*P. spumarius* has been recognized as the principal vector of *X. fastidiosa* in the Italian outbreak [12,18,19]. *P. spumarius* is widely distributed in Italy and extremely polyphagous, feeding on more than 500 plant species [20,21], thus exponentially increasing the risk of transmission of the bacterium to other plant species. Accordingly, 34 host-plant species have been found to be infected by the CoDiRO strain to date [22]. A further issue to consider is the vector capability of moving over long distances. Spittlebugs are generally considered poor fliers, notwithstanding a mark–release–recapture (MRR) study on adults of *N. campestris*, which demonstrated the ability of these insects to cover a maximum distance of 2.47 km from the release point in 35 days, and a flight capacity of almost 1.4 km in an 82 min single flight using a flight mill [23]. Conversely, Bodino et al. [24] estimated a maximum dispersal of *P. spumarius* within 400 m in a two-month trial. However, the occurrence of “hitchhiker” insect vectors has also been suggested [25,26], extending the possibility of contagion risk to even greater distances. It is therefore clear how the spread of this threat must also be constantly monitored in the nearby Italian regions, especially in those with a strong olive vocation. For instance, Calabria is the second Italian region, after Puglia, in olive-tree cultivation—184,540 vs. 382,180 ha, and in olive production—552,1476 vs. 637,3450 Mg [27]; the minimum distance between Calabria and Puglia is about 37 km, and it is just over 100 km from the outbreak area. With this in mind, the aim of this work was to implement a molecular survey on the principal pathogenic vectors of *X. fastidiosa* in the upper Ionian coasts of Calabria in order to assess the possible presence of the bacterium.

## 2. Materials and Methods

### 2.1. Vector Insect Collection and Identification

**Figure 1:**
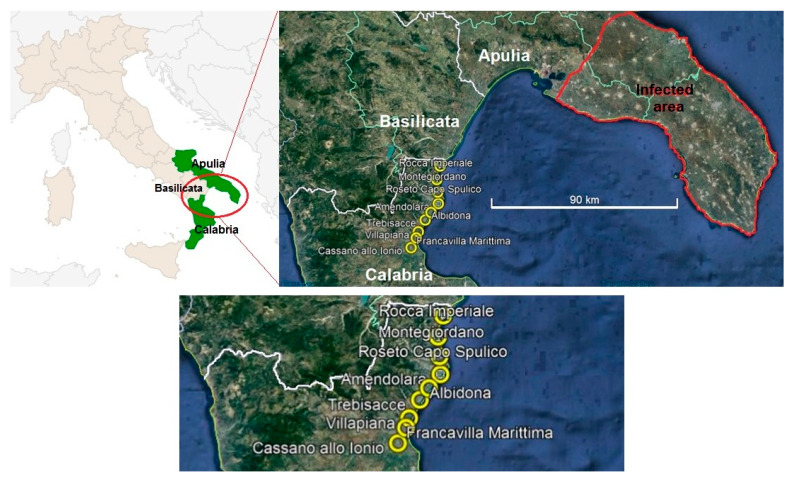
Sampling points in olive orchards or meadows alongside the main road connecting Apulia and Calabria.

Adults of spittlebugs were collected by means of sweep-net sampling within olive orchards and meadows (as biological reservoirs of entomofauna) that border Calabria’s main route of communication with Apulia, and lie in close proximity to rest areas and hotels in the upper Ionian coasts of Calabria during the August–October 2020 period. A total of 19 suitable sampling areas were chosen, falling within the municipalities of Albidona, Amendolara, Francavilla Marittima, Montegiordano, Rocca Imperiale, Roseto Capo Spulico, Cassano allo Ionio, Trebisacce, and Villapiana (Figure 1).

**Figure 2:**
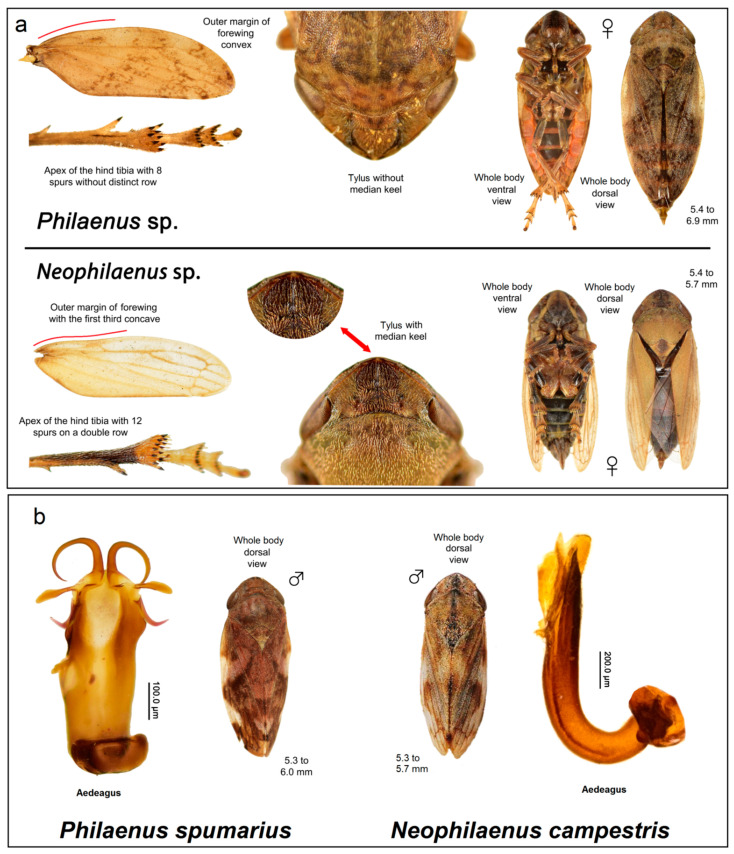
Morphological identification of the spittlebugs at the genus (**a**) and species (**b**) levels.

Once captured, the spittlebugs were stored at −20 °C and then observed under the stereomicroscope for their identification and the physical separation of the insect’s head together with the esophagus from the rest of the body (Figure 2), as described by Purcell et al. [28]. Taxonomic identification was realized by examining the morphological characteristics and male genitalia using the dichotomous key presented in [29]. 

Five heads extracted from insects assigned to the same species, collected in the same sampling date and location, were grouped together in a 2 mL tube and treated as a single sample for DNA extraction, in accordance with EPPO “PM 7/24 (4) *Xylella fastidiosa*” [30]. 

### 2.2. Statistical Analysis

A parametric one-way analysis of variance (ANOVA) was performed, after having tested the normality and homoscedasticity of residuals to assess significant differences in the number of individuals per species, according to the sampling area. A post hoc multiple comparison of means at confidence levels of 95% and 99% were made using Tukey’s honestly significant difference (HSD) test through the PAST software v.2.12.

### 2.3. Detection of X. fastidiosa in Vector Insects

DNA extraction was carried out using the CTAB (hexadecyltrimethylammonium bromide) protocol. Briefly, two tungsten carbide beads were inserted into each tube and the heads were mechanically disrupted with a Qiagen TissueLyser II (Qiagen Inc, Hilden, Germany). An amount of 500 μL of CTAB buffer (20 g of CTAB/L, 1.4 M NaCl, 0.1 M Tris/HCl, and 20 mM EDTA) was added and the solution was mixed and incubated at 65 °C for 30 min. A total of 500 μL of chloroform:isoamyl alcohol 24:1 was added and the sample was centrifuged at 16,000× *g* for 10 min. In total, 400 μL of the supernatant were added to 280 μL of cold isopropyl alcohol. The mixture was incubated at −20 °C for 20 min and centrifuged at 16,000× *g* for 20 min. The supernatant was discarded, and the DNA pellet underwent two washing phases with 1 mL of 70% ethanol, was vacuum dried, and eventually suspended in 80 μL of TE buffer (10 mM Tri-HCL, pH 8, 1 mM EDTA). DNA quality and concentration were checked for amplification with a NanoDrop 2000 spectrophotometer (Thermo scientific, Waltham, MA, USA) via end point PCR with the primer pair RST31 (GCGTTAATTTTCGAAGTGATTCGATTGC) RST 33 (CACCATTTCGTATCCCGGTG) [31], coding for a 733bp sequence of the bacterial *rpoD* gene. The reaction mixture was composed of 10 ng of template DNA, in a total volume of 25 μL containing 1 × Taq DNA polymerase buffer (Invitrogen, Carlsbad, CA, USA), 1.5 mM MgCl_2_, 200 μM dNTPs mixture, 0.3 μM forward and reverse primers, and 0.03 U μL^−1^ Taq DNA polymerase (Invitrogen, Waltham, MA, USA). The following thermal profile was used: one initial cycle at 95 °C for 1 min, 40 cycles of 95 °C for 30 s, 55 °C for 30 s, 72 °C for 45 s, and a final extension at 72 °C for 5 min [32]. At the end of the amplifications, 6 μL aliquots of each sample were subjected to horizontal electrophoresis in 1% agarose gel in TAE buffer (1.04 M TRIS–acetate, 0.002 M EDTA) after the addition of the dye. DNA from an inactivated in vitro culture of the *X. fastidiosa* strain CoDiRO was used as a positive control to evaluate the validity of the amplification. 

## 3. Results and Discussions

### 3.1. Insect Identification

A total of 563 captured spittlebugs were morphologically identified at the genus (number of apical spines on the tibia, presence/absence of median kneel, body shape; Figure 2a) and species levels (aedeagus analysis; Figure 2b) as belonging to *Philaenus Spumarius* and *Neophilaenus campestris*. This allowed us to ascertain the presence of the principal vectors of *X. fastidiosa* even in Calabria.

**Figure 3:**
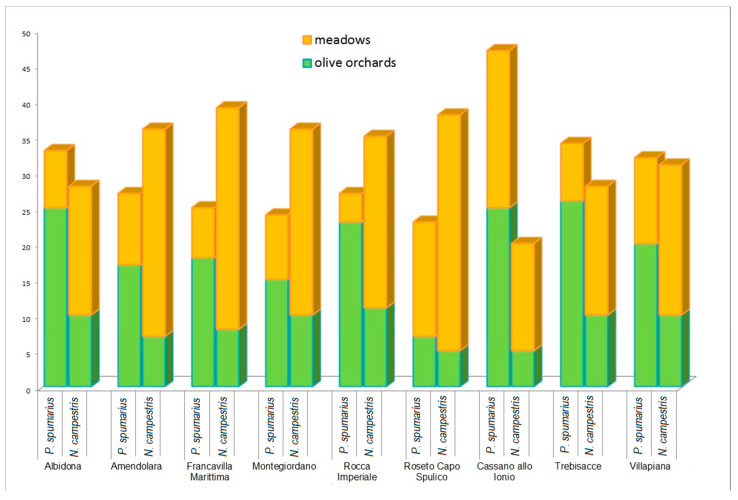
Abundance and distribution of *P. spumarius* and *N. campestris* throughout the sampling areas (municipalities).

**Figure 4:**
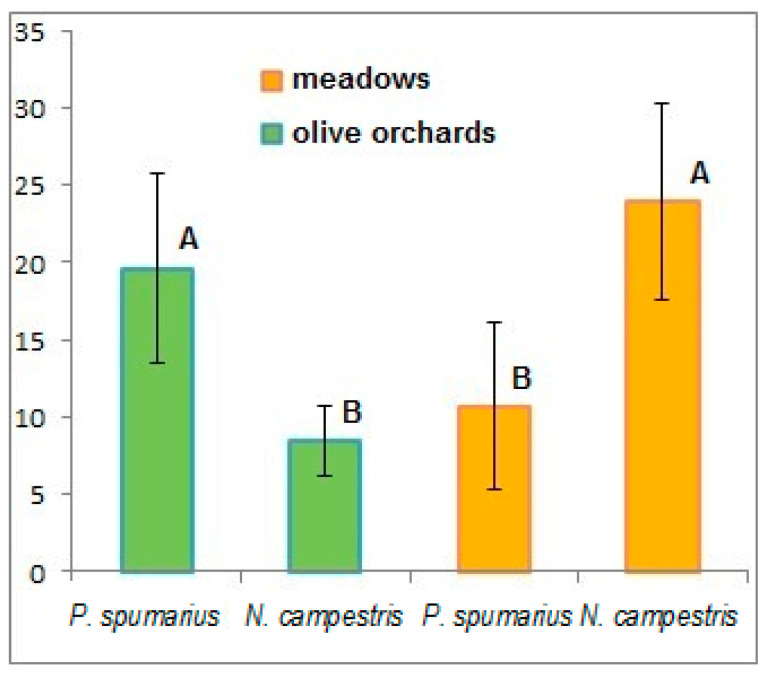
Number of individuals per species according to the sampling area (olive orchards or meadows). Different capital letters indicate a statistical difference at *p* < 0.01.

Regarding the overall relative abundance of the captured insects, *N. campestris* was the most numerous species during the open-field samplings with 321 individuals, versus 242 specimens of *P. spumarius* (Figure 3). This was due to the higher number of samplings performed in meadows where 311 (out of 563) insects were collected. In fact, a clear distinction can be highlighted between the samplings carried out in the olive groves where *P. spumarius* was found to be the prevailing species (~68%), and those performed in the meadows where *N. campestris* was the most abundant species (~77%), in accordance with previous results obtained in Apulian olive groves [19,21]. These differences were statistically significant at *p* < 0.001 (Figure 4). Both species were found to be ubiquitous and the different distributions among the municipalities can essentially be traced back to the size of the sampling area.

There was no individual of *P. italosignus* collected. This is likely due to the fact that the nymph of *P. italosignus* is monophagous on lily (*Asphodelus ramosus* L. = *aestivus* = *microcarpus*) so that it is confined to areas where its host plant is present [33]. 

### 3.2. Molecular Analysis of the Spittlebugs

**Figure 5:**
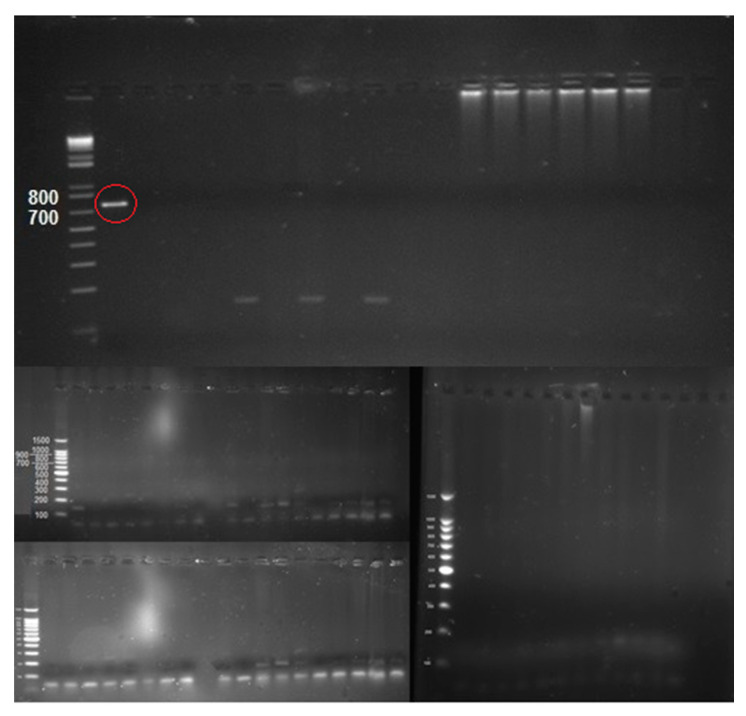
1% Agarose Gel Electrophoresis. All the samples tested negative for *X. fastidiosa*. The band in the second well, circled in red, is the positive control (DNA from an inactivated in vitro culture of *X. fastidiosa* CoDiRO strain).

The observation of the amplification products by PCR demonstrated that all 563 insects collected tested negative to *X. fastidiosa* (Figure 5). Although this result does not necessarily exclude the presence of the bacterium in the sampled area, it must be underlined that no olive grove showed phytosanitary symptoms attributable to the OQDS infection, insofar as symptoms appear when it is probably too late to intervene. However, the demonstrated natural abundance of *P. spumarius* and *N. campestris* in olive orchards and meadows, and the possibility of infected spittlebugs being hitchhiked during the summer season by tourists or commercial transporters, make the Calabrian high Ionian coast the most accessible gateway along the main communication route with Apulia. Therefore, further checks as well as active and constant monitoring are desirable for an effective fight against the advancement of the bacterium and the disease.

## 4. Conclusions

In the face of such an aggressive pathogen, it is necessary to detect and constantly monitor the most representative vectors for each area in order to promptly intervene and avoid further propagation in uncontaminated territories. Monitoring and information exchange are thus essential to build a levee against an uncontrolled spread of the infection. Furthermore, rapid methods for the molecular diagnosis of vectors permit the preventative assessment of disease containment measures before the infection of adult olive trees. Eventually, it will be necessary to activate an integrated control of the vector insects that favor agronomic and phytoiatric measures with a low environmental impact.

## Data Availability

Not applicable.
